# Langerhans-cell histiocytosis (LCH) a presentation of two siblings with two different entities

**DOI:** 10.1186/s40064-015-1139-8

**Published:** 2015-07-16

**Authors:** Mohammed Joudi Aboud, Manal Mohammed Kadhim

**Affiliations:** Unit of Pediatric Surgery, Al Diwaniya General Teaching Hospital, Al Qadisiya, Iraq; Medical Microbiology and Clinical Immunology, College of Medicine, Al Qadisiya University, Al Qadisiya, Iraq

## Abstract

Langerhans cell histiocytosis (LCH) as a term encompasses eosinophilic granuloma and two clinical syndromes: Letterer–Siwe disease and Hand–Schüller–Christian. All these syndromes seem to represent similar processes in which the proliferating cells have the structural and functional features of Langerhans cells. In reviewing the literature found that this disease does indeed occur in some families more often than would be expected. We present two boys, siblings with LCH in a single family with two different entities (There were no other siblings) and this paper is presented to emphasize the very rare familial occurrence of EG and the other one with Letterer–Siwe disease in our single family. The first patient, first child for consanguineous parents, 15 month boy, was born following an uncomplicated cesarean section, fullterm pregnancy. He developed well on bottle feed. At 13 months of age, he was noted to have a low hemoglobin concentration, mild fever, painful tender swellings and redness over the left lower chest wall a small subcutaneous mass was found. After clinical, radiological and histopathologic review, it was diagnosed as eosinophilic granuloma. Their second child, 6 month boy after a second cesarean section. At 5 month of age he developed a scaly, erythematous rash on his back spread to his shoulders, limbs and upper chest wall. The diagnostic conclusion from the clinical, skin biopsy, histopathology and bone marrow study was histiocytosis X and the diagnosis of Letterer–Siwe disease was established. Many studies listed a number of reported families with a disease which, though not considered as Letterer–Siwe disease by the authors, must certainly belong to the histiocytosis group. Many families reported have had more than one child affected with the generalized form of histiocytosis X. No case did a known relative other than a sibling have a similar disease, one family with known consanguinity, the parents were cousins. No such previous report presented these two rare different entities in two siblings as in our family.

## Background

Langerhans cell histiocytosis (LCH) as a term encompasses eosinophilic granuloma and two clinical syndromes: Letterer–Siwe disease and Hand–Schüller–Christian. These entities enrolled in similar processes in which the proliferating cells have the structural and functional features of Langerhans cells. In fact these three basic conditions represent clinical stages of the same process, but they differ in their proliferating properties, ranging from a solitary focus (eosinophilic granuloma) to disseminated multifocal skeletal (Hand–Schuller–Christian) and disseminated multifocal skeletal and extraskeletal disease (Letterer–Siwe disease). Eosinophilic granuloma (EG) or benign focal histiocytosis-X is one of these clinical entities, which involves the reticuloendothelial system known as histiocytosis-X, and was reported in 1953 (Willets et al. 1998). EG frequently occurs in the flat bones such as skull, pelvis, scapula, and the ribs (Raijman 1998). Many modalities of treatment has been reported for solitary eosinophilic granuloma of bone, including observation, injections of steroid, local excision and curettage with or without bone grafting, chemotherapy and irradiation. All of these treatments are reported to give satisfactory results with a recurrence rate of less than 20% (Ioannidis et al. 2011; Stull et al. 1992; Satter et al. 2008; Bechan et al. 2006). The age of the patients in these series varies widely. LCH in its other systemic manifestations includes Hand–Schüller–Christian disease with a triad of exophthalmos, diabetes insipidus and osteolytic lesions of the skull, and Letterer–Siwe disease with skin rash, fever, lymphadenopathy, hepatosplenomegaly, anaemia and thrombocytopenia (Lichtenstein 1964). In reviewing the literature found that this disease does indeed occur in some families more often than would be expected (Falk and Gellei 1957).

We present two boys, siblings with LCH in a single family with two different entities (there were no other siblings), managed at the pediatric surgery unit, the maternity and Child Teaching Hospital, Al Qadisiya, Iraq, this paper is presented to emphasize the very rare familial occurrence of EG and the other one with Letterer–Siwe disease in our single family. The clinical course in these two infants is described. Written informed consent was obtained from the parents for publication of the reported cases and any accompanying images.

## Case description

### Case 1

**Figure 1:**
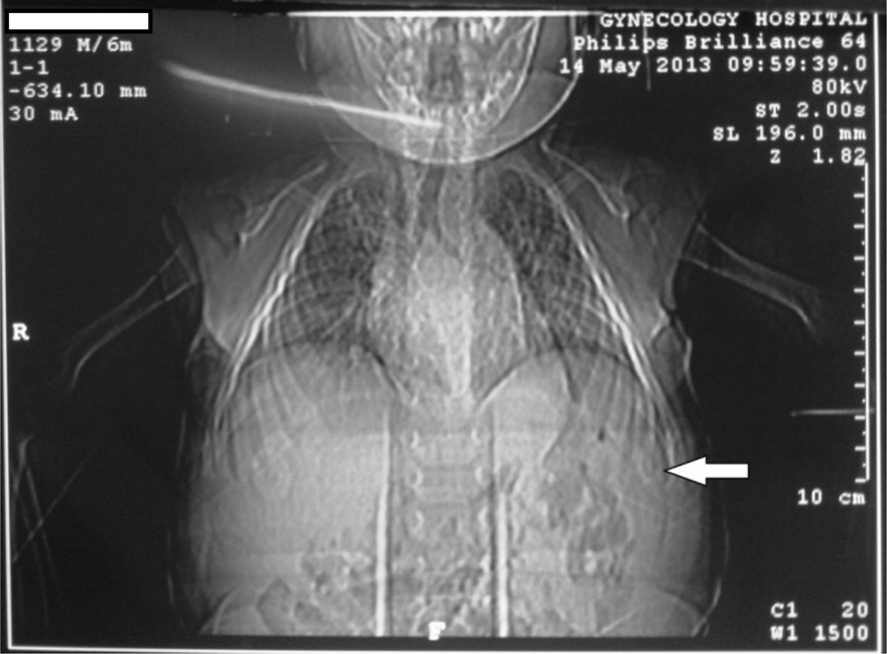
Plain chest radiograph showed an irregular 20 × 10 mm lytic expansile lesion (*arrowed*) involve and covering the anterior angle of the 8th rib.

**Figure 2:**
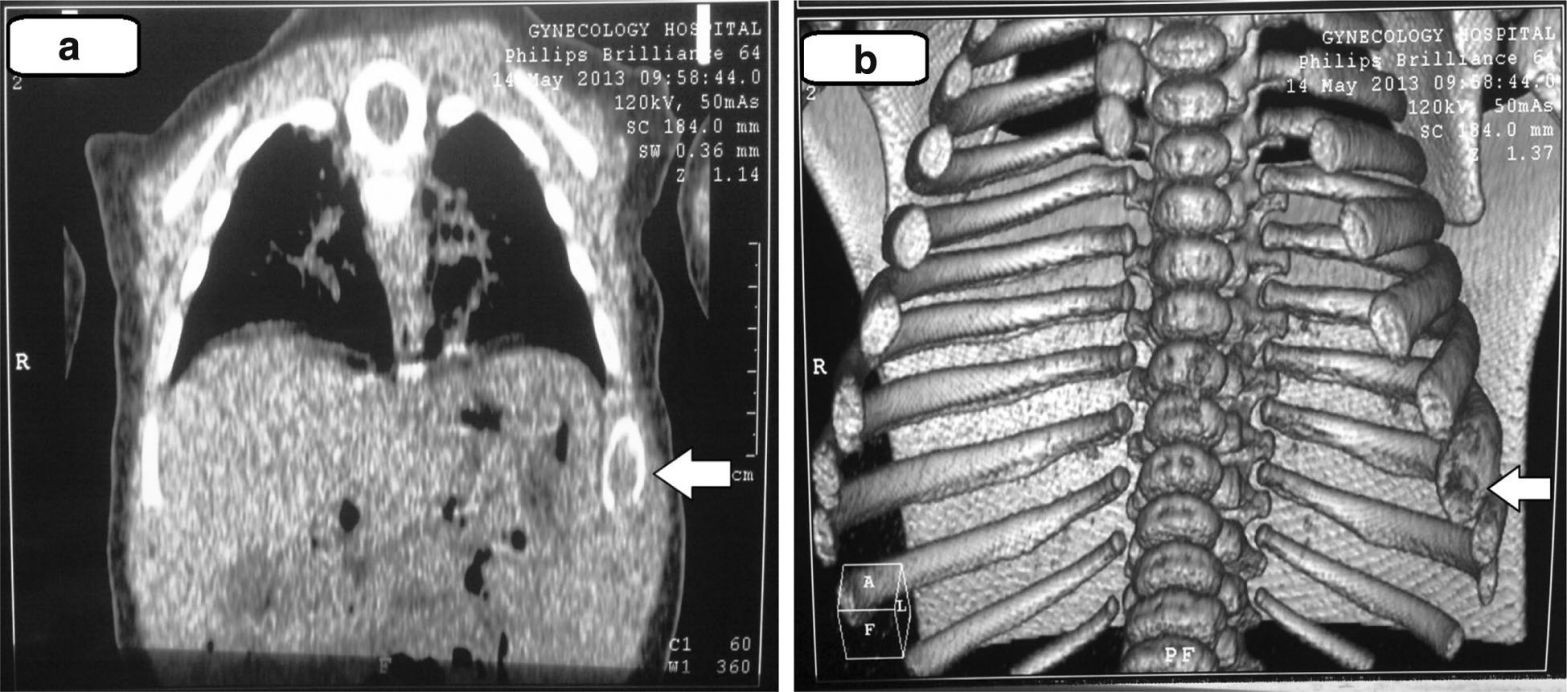
Chest computed tomography native (**a**), three dimensional (**b**), showed the lateral part of the left 8th ribs with expansile lytic changes with breaching out and some surrounding soft tissue components.

**Figure 3:**
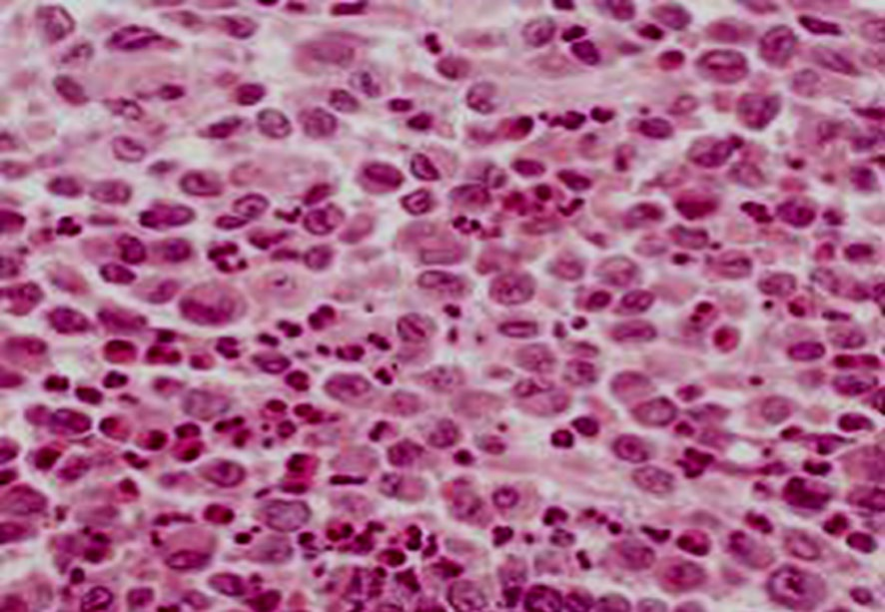
Hematoxylin-eosin × 100 stained sections demonstrated a sheet of Langerhans cells mixed with variable numbers of eosinophils.

The first patient, first child for consanguineous parents, 15 month boy, was born following an uncomplicated cesarean section, full-term pregnancy. He developed well on bottle feed. At 13 months of age, he was noted to have a low hemoglobin concentration, mild fever, painful tender swellings and redness over the left lower chest wall a small subcutaneous mass was found. The parents had no idea when they first felt the lesion but they had the impression it was slowly increasing over time. Blood profile revealed slightly elevated erythrocyte sedimentation rate (33 mm/h) and other laboratory examination was within the normal limits. The plain chest radiograph showed an irregular 20 × 10 mm lytic expansile lesion involve and covering the anterior angle of the 8th rib with cortical breach, no soft tissue extension, no calcification and clear both lungs (Figure 1). Fibrous dysplasia, eosinophilic granuloma, osteolytic metastasis, myeloma, chondroma, and osteomyelitis were considered as in differential diagnosis. Chest computed tomography native showed the lateral part of the left 8th ribs with expansile lytic changes with breaching out and some surrounding soft tissue components (Figure 2a, b). No facilities for nuclear bone scanning in our center. Fine needle aspiration biopsy was performed from the well defined left lytic lesion and the specimen was submitted for histopathologic examination to establish a diagnosis and treatment plan, it revealed a few groups of benign looking mesenchymal cells, admixed with inflammatory cells including many eosinophils, no evidence of malignant cells. After histopathologic review, it was diagnosed as eosinophilic granuloma. Microscopically, the hematoxylin-eosin stained sections demonstrated a sheet of Langerhans cells mixed with variable numbers of eosinophils (Figure 3). Immunohistochemical staining showed that the histiocytic cells were positive for the S-100 proteins and strong positivity for CD1a. Due to the small size of the pathology, nature of the lesions, the risk of deformity and pathologic fracture was minimal, observation strategy was applied and the progress of pain was decreasing with analgesia and steroid, prednisone 40 mg per week tapered gradually to 10 mg. Six months after treatment, pain has fully disappeared. The patient was followed radiologically for 2 years and all the signs and the mass have improved and subsided spontaneously. A his last consultation, the baby was completely asymptomatic.

### Case 2

**Figure 4:**
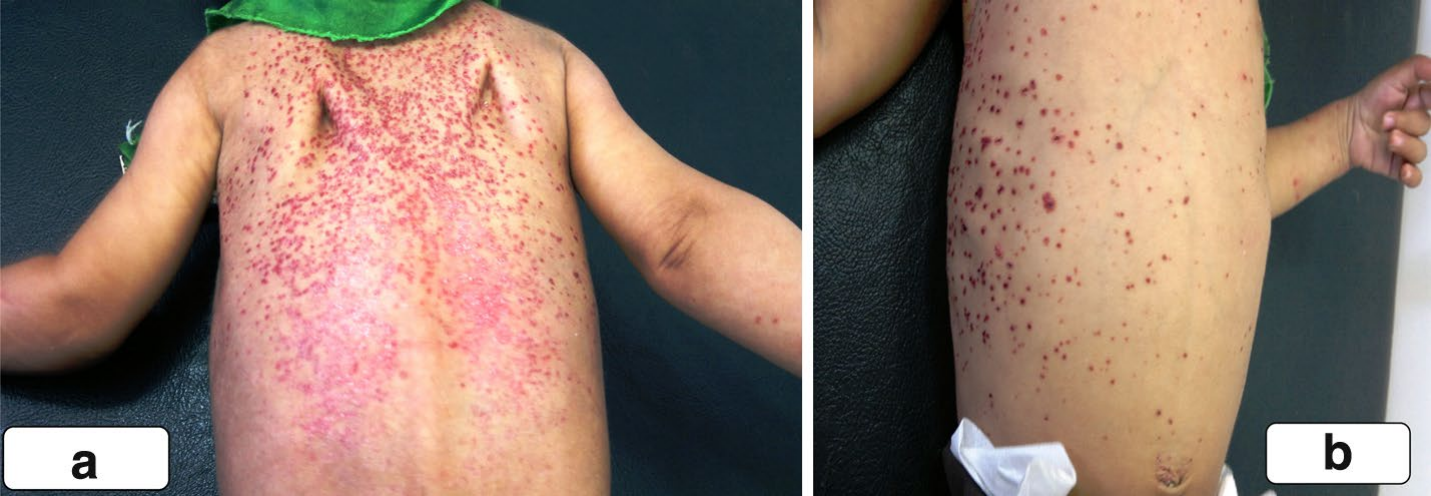
A scaly, erythematous rash on the back of the second boy spread to his shoulders (**a**) and upper chest wall (**b**).

**Figure 5:**
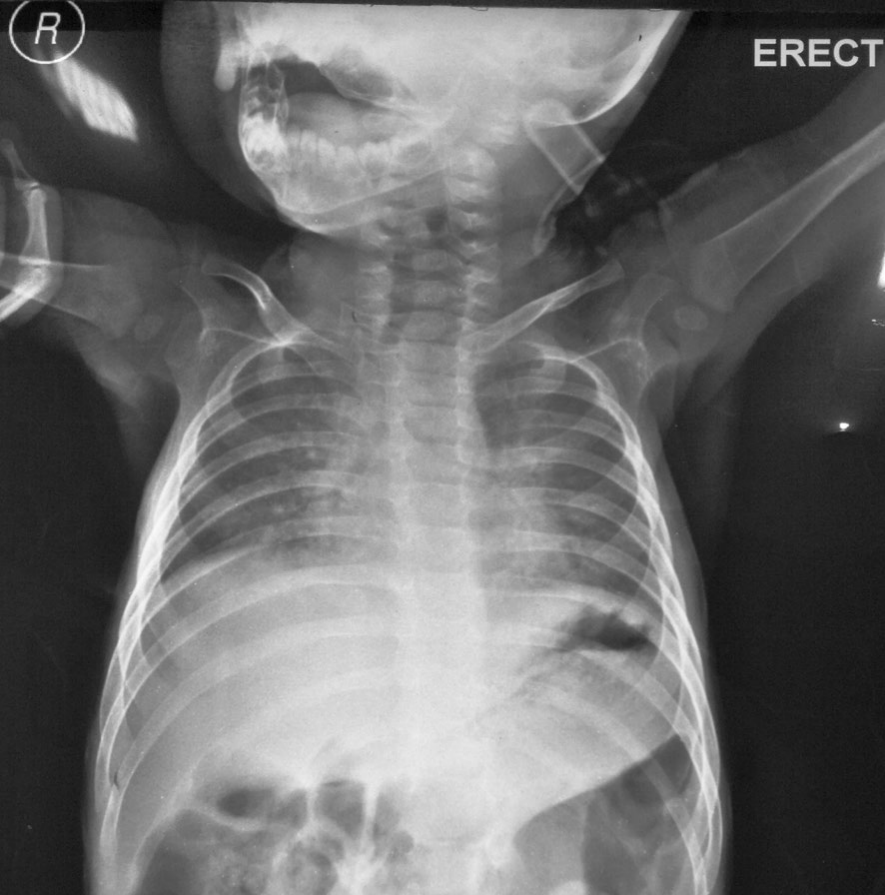
Plain chest X-ray film suggested cardiomegaly with increased interstitial markings and upper mediastinal lymph nodes enlargement.

**Figure 6:**
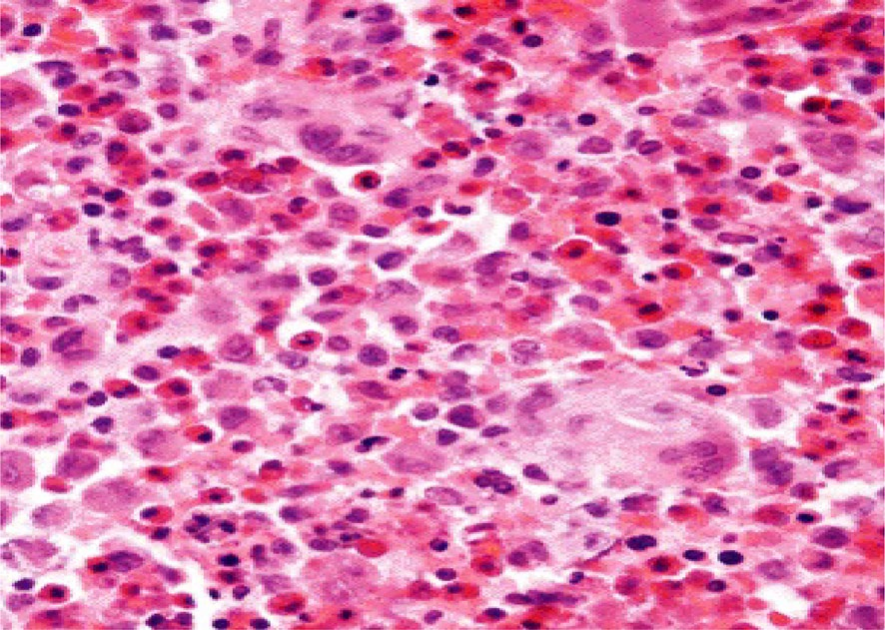
Skin biopsy histopathology (hematoxylin and eosin × 100), revealed aggregates of histiocytic-type cells with an abundant eosinophilic and granular cytoplasm. Immunohistochemical studies done, epidermal LCs for CD1a reactivity noticed.

Their second child, 6 month boy after a second cesarean section. At 5 month of age he developed a scaly, erythematous rash on his back spread to his shoulders, limbs and upper chest wall (Figure 4a, b). The rash recurred after short period of response on to topical steroids. Two weeks before presentation he developed vomiting post feeding and meal, mild fever, cough, tachypnea, anemia and he lost 400 g in weight during last 3 weeks before presentation. Initial clinical evaluation started on admission with team approach strategy, blood parameters and biochemistry results revealed wight 5.2 kg, hepatosplenomegaly (live 2 cm palpable below costal margin), lymphadenopathy, anemia Hb 8.9 g/100 ml and thrombocytopenia, a platelet count of 66,000/mm^3^ and elevated erythrocyte sedimentation rate (48 mm/h). A plain chest X-ray film suggested cardiomegaly with increased interstitial markings and upper mediastinal lymph nodes enlargement (Figure 5). Skull and long bones were normal. Skin biopsy suggested and the histopathology revealed ill defined cell margins, the diagnostic nuclei were bean shaped with nuclear grooves and indentations, collections of reticulum cells in the dermis with thinning of the overlying epidermis and aggregates of histiocytic-type cells with an abundant eosinophilic and granular cytoplasm. Immunohistochemical studies done, epidermal LCs for CD1a and S-100 protein reactivity were noticed (Figure 6). The result of bone marrow aspiration was some reduction of platelets and considerable reduction of granulocyte formation, a pronounced normoblastosis, with some cytoplasmic nuclear dissociation, no clear evidence of malignant changes in the normoblastic series. There was marked proliferation of reticuloendothelial cells. Multinucleated cells were seen frequently and frank giant cells occasionally. The diagnostic conclusion from the bone marrow study was histiocytosis *X* and the diagnosis of Letterer–Siwe disease was established as the family had a little bit such scenario before 2 years ago. Two days after admission medication started with antibiotics and steroid, Prednisolone 5 mg/kg per 24 per day. Poor facilities declined anti metabolite medication and his condition did not accept transfer to sophisticated oncology center. Two weeks after admission, in spit the skin rash decreased and the fever subsided, the baby became increasingly breathless and all evidence of cardiac and respiratory failure were rapidly developed, anti failure measurement started, oxygen, diuretics and digoxin. After 3 weeks post admission he passed in unfortunate fate, died, and for medico legal documentations, our authority policies do not allow the necropsy role.

## Discussion

In term LCH represents a childhood disease and the others multisystem forms of the disease, formerly called Hand–Schüller–Christian and Letterer–Siwe disease (Ioannidis et al. 2011), appear almost exclusively in children between 1 to 5 years old and younger than 2 years, respectively (Stull et al. 1992). Eosinophilic granuloma is usually seen in children 5–15 years old (Stull et al. 1992) and seldom in adults. The etiology and pathogenesis of the disease still remains unknown. In some literatures have been reclassified as single organ system disease, unifocal or multifocal, and multisystem disease, complicated or not by organ dysfunction (Satter and High 2008). A clinicopathologic results offers the diagnosis, based on classical findings of clinical and histologic/immunohistochemical criteria. Reactive normal Langerhans cells found within nodes, to avoid misdiagnosis, they may present as a response to a variety of diseases including neoplasms (Bechan et al. 2006; Beverley et al. 2005). In literatures all the forms of LCH are characterized by clonal proliferation of CD1+ histiocytes and the absolute criteria for diagnosis depended on finding CD1a by immunohistochemistry or Birbeck granules by electron microscopy. Positivity of one or both of these finding now defines the Langerhans cell phenotype (Romani et al. 2003; Sidler et al. 2000; Chu 2001). Despite infections, immune and neoplastic causes have been postulated (Moon et al. 2009; Plasschaert et al. 2002), the pathogenesis is not completely understood. Near 80% of histiocytosis X lesions in children are of solitary EG type and about 90% occur in children (Jayaprakash et al. 2012). Usually Letterer–Siwe disease occurs in infants less than 2 years old and presents clinical symptoms such as fever, skin rush, anemia, hepatosplenomegaly, lymphadenopathy, and bleeding diathesis. The clinical course is rapid and fatal. Despite the tropism of skin lesions to flexures also suggests that external stimuli may trigger inflammation (Weitzman and Egeler 2008). However, the nature of the initiating event(s), and the mechanisms of local tissue destruction by LCH and other inflammatory cells are still largely unknown.

Until the recent time the specific genetic abnormalities of LCH were not consistently observed, in one study a genetic component is suggested by a higher concordance rate between monozygotic twins compared with dizygotic twins (Arico et al. 1999). Many studies listed a number of reported families with a disease which, though not considered as Letterer–Siwe disease by the authors, must certainly belong to the histiocytosis group (Freundlich et al. 1972). Juberg et al. 1970 suggested by analyzing the occurrence of Letterer–Siwe disease in monozygotic twins, siblings, and consanguineous matings that this disease results from an autosomal recessive gene with low penetrance (Satoshi and Masaru 1990). Clonality of LCH granulomas has been reported in 1994 (Willman et al. 1994; Yu et al. 1994). Takashi et al. (2012) described a mutant alleles in some family groups and he mentioned specifically his finding with the clinical features of patients with B-RAF mutations V600EB-RAF mutations were found both in children with granuloma of bones or isolated skin disease, and in infants with early-onset multi-organ disease and he mentioned that the somatic mutation in some patients confirmed despite the mother with the same allele, was in good health and did not report a personal history of LCH. This is the same story with our cases but unfortunately we have no such facilities to study the precise genetic background of the family. Recently some studies considered eosinophilic granuloma and Hand–Schuller–Christian disease as a single disease entity (Satoshi and Masaru 1990; Forssman and Rudberg 1960), many investigators describe Letterer–Siwe disease as an entity distinct from eosinophilic granuloma and Hand–Schuller–Christian disease as the disease considered to be a malignant, fulminant lymphomatous disease of infants. Important study mentioned to good support that Hand–Schuller–Christian disease and eosinophilic granuloma are different clinical expressions of the same disease entity, despite the rarity in familial occurrence, while it is frequent in Letterer–Siwe disease (Satoshi and Masaru 1990; Vogel and Vogel 1972). Many families reported have had more than one child affected with the generalized form of histiocytosis X. Some of them had three affected children, others families have each had two affected children. No case did a known relative other than a sibling have a similar disease, one family with known consanguinity, the parents were cousins (Volker 1963).

## Conclusion

No such previous report presented these two rare different entities in two siblings as in our family. As the background of all modalities of LCH may enrolled in the same process, further studies suggest to verify and obtain a precise data about the genetic, cellular, immunological, environmental and clinical profile of pedigree or parent with diseased sibling.

## Endnote

We are grateful for the editors to review this article managed at the Maternity and Child Teaching Hospital. The Study conducted on the terms and policy of the institution and the College of Medicine, they announced their agreement for participants, a printed, optical, electronic document and database designed to record all of the written information. The rarity of such presentation made our decision to declare the cases, hope to have more precise study and investigation with qualified resources to obtain more knowledge about the occurrence of the disease among the family siblings.
